# Targeted Next-Generation Sequencing at Copy-Number Breakpoints for Personalized Analysis of Rearranged Ends in Solid Tumors

**DOI:** 10.1371/journal.pone.0100089

**Published:** 2014-06-17

**Authors:** Hyun-Kyoung Kim, Won Cheol Park, Kwang Man Lee, Hai-Li Hwang, Seong-Yeol Park, Sungbin Sorn, Vishal Chandra, Kwang Gi Kim, Woong-Bae Yoon, Joon Seol Bae, Hyoung Doo Shin, Jong-Yeon Shin, Ju-Young Seoh, Jong-Il Kim, Kyeong-Man Hong

**Affiliations:** 1 Research Institute, National Cancer Center, Ilsandong-gu, Goyang, Korea; 2 Department of Microbiology, Ewha Womans University School of Medicine, Seoul, Korea; 3 Department of Surgery, Wonkwang University School of Medicine, Iksan, Korea; 4 Laboratory of Translational Genomics, Samsung Genome Institute, Samsung Medical Center, Seoul, Korea; 5 Department of Life Science, Sogang University, Seoul, Korea; 6 Department of Genetic Epidemiology, SNP Genetics, Inc., Seoul, Korea; 7 Genomic Medicine Institute, Medical Research Center, Seoul National University, Seoul, Korea; 8 Department of Biomedical Sciences, Seoul National University Graduate School, Seoul, Korea; 9 Department of Biochemistry and Molecular Biology, Seoul National University College of Medicine, Seoul, Korea; UT MD Anderson Cancer Center, United States of America

## Abstract

**Background:**

The concept of the utilization of rearranged ends for development of personalized biomarkers has attracted much attention owing to its clinical applicability. Although targeted next-generation sequencing (NGS) for recurrent rearrangements has been successful in hematologic malignancies, its application to solid tumors is problematic due to the paucity of recurrent translocations. However, copy-number breakpoints (CNBs), which are abundant in solid tumors, can be utilized for identification of rearranged ends.

**Method:**

As a proof of concept, we performed targeted next-generation sequencing at copy-number breakpoints (TNGS-CNB) in nine colon cancer cases including seven primary cancers and two cell lines, COLO205 and SW620. For deduction of CNBs, we developed a novel competitive single-nucleotide polymorphism (cSNP) microarray method entailing CNB-region refinement by competitor DNA.

**Result:**

Using TNGS-CNB, 19 specific rearrangements out of 91 CNBs (20.9%) were identified, and two polymerase chain reaction (PCR)-amplifiable rearrangements were obtained in six cases (66.7%). And significantly, TNGS-CNB, with its high positive identification rate (82.6%) of PCR-amplifiable rearrangements at candidate sites (19/23), just from filtering of aligned sequences, requires little effort for validation.

**Conclusion:**

Our results indicate that TNGS-CNB, with its utility for identification of rearrangements in solid tumors, can be successfully applied in the clinical laboratory for cancer-relapse and therapy-response monitoring.

## Introduction

Tumor-specific, widespread rearrangement of DNA is a universal feature of cancer. Because rearrangement is not present in normal cells, it can be a useful means of monitoring cancer relapse and response to therapy [1], [2], [3]. Initially, the recurrent rearrangements including *BCR*-*ABL*, *AML1*-*ETO*, *TEL*-*AML1*, and *TML*-*RARA* were used with conventional technologies such as reverse-transcription polymerase chain reaction (RT-PCR) to monitor minimal residual tumors and classify hematologic malignancies [1], [4], [5]. Their clinical implications, in the context of hematologic malignancies, subsequently has been confirmed by several studies [6], [7], [8], [9]. Recurrent rearrangements, however, are rare in solid tumors, and in most cases, information on rearranged sequences is not available.

Recently, whole-genome next-generation sequencing (NGS) has been employed to obtain information on rearranged sequences, and its clinical application in cancer has been successfully demonstrated [10], [11]. Although the acquisition of NGS data by now is relatively straightforward, its analysis can be extremely complicated and time consuming, due to data volumes and computational difficulty in aligning short reads [12], [13]. To circumvent these problems, a targeted-capture method in combination with NGS for 20 genes showing recurrent translocation has been applied to identify translocations in leukemia [13]. However, the application of targeted NGS to solid tumors is impractical, due simply to the paucity of recurrent translocations. Alternatively, and given that copy-number breakpoints in solid tumors contain cancer-specific translocations [14], [15], in the present study, we performed a mode of targeted next-generation sequencing at copy-number breakpoints (TNGS-CNB). To obtain the copy-number breakpoints, we used a novel competitive single-nucleotide polymorphism (cSNP) microarray method incorporating competitor DNA from hydatidiform-mole (H-mole) cells to obtain more refined sequence information, and designed targeted-capture probes to enrich candidate rearranged sequences. For cost-reduction ends, we employed a single capture probe set (instead of nine) for nine samples consisting of seven primary colon cancer tissues and two colon cancer cell lines, COLO205 and SW620.

## Materials and Methods

### Cancer Tissues and Cell Lines

The use of fresh-frozen colon cancer tissues, corresponding normal colon tissues, and control blood-DNA samples was approved by the Institutional Review Boards of both the National Cancer Center and Wonkwang University School of Medicine. SW620 and COLO205 cell lines were obtained from the National Cancer Institute (MTA No. 2702-09). Human H-mole-cell DNA was purchased from Coriell (NA07489, Camden, NJ). The Institutional Review Boards waived the need for informed consent from patients whose samples were taken before 2005, according to the Enforcement Decree of Bioethics and Safety Act in Korea.

### DNA Isolation

DNA from the frozen colon cancer tissues and cancer cell lines was isolated using the DNeasy Blood and Tissue Kit (Qiagen, Valencia, CA) after 12 hr incubation at 55°C in 100 mM Tris, pH 8.0 buffer containing 1% SDS, 5 mM EDTA, 10 mM NaCl, and 500 µg/ml proteinase K. DNA was extracted from 10 to 20 sections (10 µm thickness) of each fresh-frozen tissue. The contents of the cancer cells in the fresh-frozen cancer tissues were assessed on H&E-stained tissue-section slides. Those containing 60% or more cancer cells were used in the present study.

### SNP Microarray Analysis

Copy-number alterations were analyzed using a CytoSNP-12 microarray containing 294,975 markers for detection of abnormalities across the genome (Illumina, San Diego, CA). Concentrations of H-mole DNA and sample DNA were analyzed by Quant-iT PicoGreen dsDNA Reagents (Invitrogen, Eugene, OR), and their equal amounts were mixed for cSNP microarray analyses.

DNA amplification, tagging, and hybridization were performed at SNP Genetics (Seoul, Korea) according to the manufacturer’s protocol for the Infinium assay® (Illumina), using an initial total DNA amount of 200 ng per microarray. The hybridized array slides were scanned on an iScan Reader (Illumina). In order to obtain information on copy alterations in the SNP microarray, the B allele frequency (BAF) and Log R ratio (LRR) were determined using the GenomeStudio software (version 2011.1, Illumina). The BAF and the LRR are the normalized measures of allelic intensity ratio and the total signal intensity ratio of two alleles, respectively, as described previously [16], [17], [18].

### Analysis of Copy-Number Breakpoints in cSNP Microarray Data

H-mole DNA was used as a competitor for a cSNP microarray analysis. The SNP microarray results for 1) H-mole cells, 2) normal colon cells, 3) the mixture of H-mole and normal colon DNAs, and 4) the mixture of H-mole and colon cancer DNAs, were employed in the analysis. In the normal-tissue or H-mole DNA, the alleles having a BAF >0.95 or <0.05 were regarded as homozygous. “Alter homozygote SNP” was defined as a homozygous allele in normal-tissue DNA, which differs from an allele in H-mole DNA. By utilizing the SNP data from the H-mole and normal-tissue DNA, only alter homozygote SNPs were extracted and employed in CNB deduction.

For the calculation of the copy numbers from the SNP microarray results, only alter homozygote SNP alleles were employed. The formulas for calculation of the allelic ratio (AR) from the BAF value at each alter homozygote SNP are as follows: AR = BAF/(1− BAF) when the normal homozygous allele is the B allele, and AR = (1− BAF)/BAF when the normal homozygous allele is not the B allele. The ARs for the mixtures of normal-tissue and H-mole DNAs (AR_NH_) and for the mixtures of cancer and H-mole DNAs (AR_CH_) were calculated from the BAF_NH_ (for the mixture of normal-tissue and H-mole DNAs, or the N-H mixture) and the BAF_CH_ (for the mixture of cancer and H-mole DNAs, or the C-H mixture), respectively. The AR_CH_/AR_NH_ ratio (the AR ratio or ARR) represents the copy status of cancer cells relative to normal cells. Further ARR normalization was necessary, due to the incurring of experimental errors during DNA mixing; that is, the ARR was divided by the mean ARR value in a specific sample, and the resulting normalized ARR (nARR) was employed to represent the copy status of that sample. Software for cSNP microarray analysis is available upon request.

In the case of the cancer cell lines, corresponding normal-tissue DNAs were not available, and so the AR_CH_ value was used in place of the ARR. After normalization by the mean AR_H&C_ value, the resulting normalized AR_CH_ (nAR_CH_) value was considered to represent the copy status of the cancer cells relative to the normal cells. Additionally, for comparison with the data for copy-number alteration by nARR, the nAR_CH_ value was analyzed also for each primary colon cancer case. The neighboring alleles showing an abrupt change in the nARR or nAR_CH_ values in a chromosome were selected as CNBs; at least five CNBs were selected for each sample.

### Targeted Capture of Rearranged Sequences

For targeted capture of CNBs, a 3 M SureSelect Target Enrichment Capture Array (Agilent Technology, Santa Clara, CA, USA) with a probe size of 120-bp was designed to 2x tile using the web-based design tool eArray (Agilent Technology). To reduce the cost, one SureSelect Capture Array was designed (instead of nine) across 91 CNBs for all nine samples, and SureSelect Capture Arrays for 16 samples, with the same probe set, were provided. The estimated size of the total capture region was 3.8 Mb; however, by removing repetitive regions, it was reduced to 2.2 Mb.

The targeted-capture procedure was performed according to the manufacturer’s protocol for the SureSelect^XT^ Target Enrichment System with the Illumina Paired-End Sequencing Library (Agilent). Approximately 3.0 µg of genomic DNA from each sample was sheared to fragments of 150–200 bp using the Covaris S2 Sonolab (Covaris, Woburn, MA) at a 20% duty cycle, level 5 intensity and 200 cycles per burst for 180 s. After the fragment ends were repaired, the paired-end adaptors were ligated. Small fragments (<100 bp) and unligated adaptors were removed by AMPure purification (Agencourt Bioscience, Beverly, MA, USA). Then, the library was hybridized with capture probes, according to the protocol. The resulting RNA probe/DNA hybrids were recovered using streptavidin-labeled magnetic beads. After removal of the cRNA probes by RNase treatment, the captured DNA fragments were amplified using universal primers.

### Next-Generation Sequencing and Data Processing

After the targeted capture for each sample, 101-bp paired-end NGS was performed with the Illumina Hiseq2000 (Illumina). The resulting FASTQ files were aligned to the NCBI human genome assembly (build 37, hg19) using the Genomic Short-read Nucleotide Alignment Program [19] with allowance for 5% mismatches as previously reported [20].

To find DNA rearrangements and their breakpoints in the targeted sequencing data, we modified the previous methods employed for detection of large-deletion breakpoints [20] and fusion genes [21]. Specifically, we first listed discordant paired-end reads for which one read was aligned to target the captured region but the other was aligned to a different chromosome or in the same chromosome but separated by a distance of more than 1 kb. Afterwards, we selected rearranged sequences wherein more than three discordant paired-end reads were mapped within a 2 kb window. To determine the breakpoints, we selected orphan read pairs in which one end was mapped near any CNB and the other was not aligned to the human genome reference sequence. Those unmapped ends were re-aligned to the reference genome using the BLAST3 program, so as to determine if it could be split and separately aligned to two CNB sites. We excluded the rearranged sequences wherein no split reads were found. After removing repetitive sequences, the reads containing rearranged sequences outside of the sample-specific CNB sites were excluded, because the SureSelect probe set for the specific samples contained probes for the other samples as well.

### Confirmation of Rearranged Sequences

For the purpose of obtaining polymerase chain reaction (PCR)-amplifiable rearranged sequences, PCR primers were designed for the rearranged sequences confirmed by targeted sequencing (Table S1), and PCR was performed for each sequence under the following conditions: initial incubation at 95°C for 10 min, followed by 35 cycles of 30 s at 95°C, 30 s at 56°C, and 1 min at 72°C in a mixture containing 1X PCR buffer II (Roche, Mannheim, Germany) with 1.5 mM MgCl_2_, 0.2 mM dNTPs, 10 pmol of each primer, and 20 ng of genomic DNA in a final volume of 20 µl. The amplified products were purified using the AxyPrep PCR Clean up kit (Axygen, Union City, CA) in order to remove leftover primers, and were then sequenced with the forward or reverse primers used in the PCR reaction (Table S2). The presence of DNA in samples was confirmed by PCR for *IGF1* using the following conditions: initial incubation at 95°C for 10 min, followed by 35 cycles of 30 s at 95°C, 30 s at 58°C, and 30 s at 72°C in a mixture containing 1X PCR buffer II (Roche) with 1.5 mM MgCl_2_, 0.2 mM dNTPs, 10 pmol of each primer for *IGF1* (Table S2), and 20 ng of genomic DNA in a final volume of 20 µl.

### Study Design for Obtainment of Information on Rearrangement Sites

**Figure 1:**
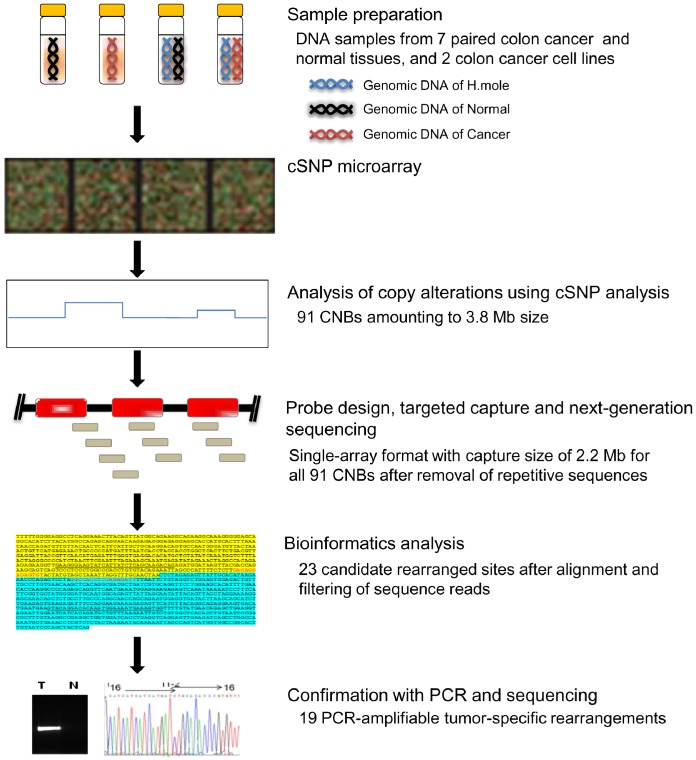
TNGS-CNB schematic. The copy-number statuses of seven primary colon cancer tissues and two colon cancer cell lines were analyzed by the cSNP microarray method, and 91 CNBs were deduced. After removing 3.8 Mb of repetitive sequences from the CNB regions, the area for targeted capture was 2.2 Mb. After paired-end NGS of the captured sequences, the reads were aligned to the NCBI human genome assembly (build 37, hg19), and 23 candidate rearranged sequences were deduced. After PCR confirmation of the candidate rearranged sequences, 19 PCR-amplifiable rearrangements were identified.

The overall procedural scheme of the present study is shown in Figure 1. Among eight primary colon cancer cases, one lacked a sufficient number of CNBs, and thus was excluded from further analysis. From the microarray copy-alteration data, 91 CNBs from the seven primary colon cancer and the two cancer-cell-line samples were selected. A sequence-capture array was designed for all 91 CNBs from the nine samples. After targeted capture, paired-end NGS was performed. Following the alignment and filtering of the sequence reads, there were 23 candidate rearrangement sites, among which 19 were confirmed by tumor-specific PCR amplification.

### Copy-Alteration Analysis by cSNP Microarray using Competitor DNA

**Figure 2:**
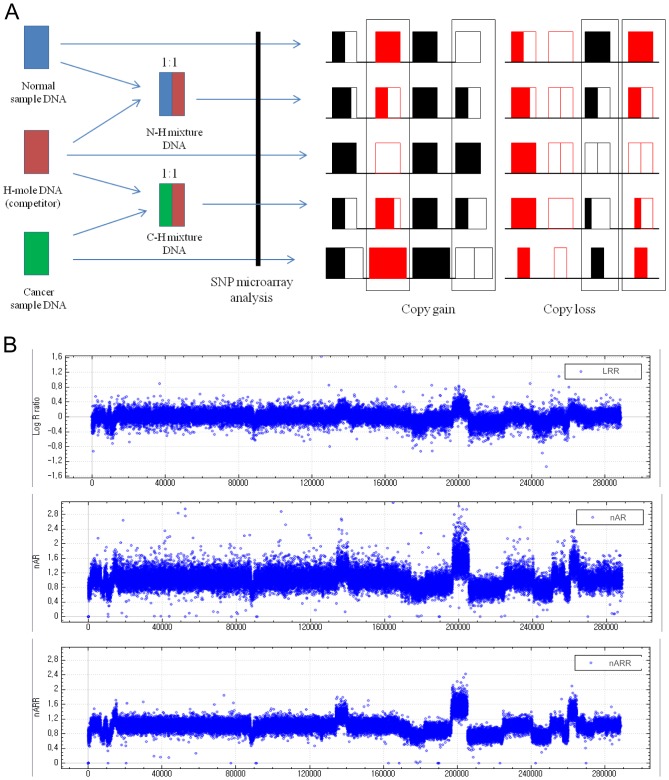
cSNP microarray for determination of copy-number breakpoints. A. Schematic procedure for cSNP microarray. For each sample, SNP microarray experiments were performed for 1) H-mole DNA, 2) normal-sample DNA, 3) the mixture of normal and H-mole DNAs (N-H mixture), and 4) the mixture of cancer and H-mole DNAs (C-H mixture). An SNP microarray experiment for cancer samples was performed for the purpose of comparison. Only alter homozygous alleles (box) were selected for the cSNP microarray analysis. B. Representative LRR, nAR_CH_, and nARR results for colon cancer samples. The LRR was obtained by Genome Studio software; the nAR_CH_ was calculated by cSNP software for the cancer and H-mole DNAs; the nARR was calculated by the same cSNP software for DNAs from the cancer tissues, corresponding normal tissues, and H-mole.

A schematic of the cSNP microarray procedure using competitor H-mole DNA is shown in Figure 2A. For each sample, SNP microarray experiments were performed for 1) H-mole DNA, 2) normal-sample DNA, 3) the N-H mixture, and 4) the C-H mixture. Only alter homozygote alleles are informative, and are employed for the determination of copy status. Among the 290 K alleles in the CytoSNP-12 microarray, alter homozygous alleles numbered about 45–50 K per sample.

To determine copy status from SNP microarray results, the nARR is derived from alter homozygote alleles’ BAF values. Under ideal conditions, the ratio of cancer to H-mole DNAs in the C-H mixture is exactly 1∶1, and the AR_CH_ is the copy status at the specific allele, as shown in Table 1. However, experimental errors are incurred in mixing two sample DNAs. When the ratios of two sample DNAs in the C-H and N-H mixtures, are the same (e.g. 2∶3, as shown in Table 1), the ARR, rather than the AR_CH,_ represents the copy status. When the ratios of two mixture samples are neither 1∶1 nor the same, only nARR can be the copy status (Table 1), indicating that only nARR can be employed for calculation of copy status in a cSNP microarray.

**Table 1:** Copy-number status information by cSNP microarray.

N-H mix ratio	C-H mix ratio	Copy Number	Cancer genotype	Normal genotype	H-mole genotype	N-H mix genotype	C-H mix genotype	BAF_NH_	AR_NH_	BAF_CH_	AR_CH_	ARR	nARR
2∶2 or 1∶1	2∶2 or 1∶1	4	BBBB	BB	AA	AABB	AABBBB	2/4	2/2	4/6	**4/2 (2.00)**	**2.00**	**2.00**
		3	BBB	BB	AA	AABB	AABBB	2/4	2/2	3/5	**3/2 (1.50)**	**1.50**	**1.50**
		2	BB	BB	AA	AABB	AABB	2/4	2/2	2/4	**2/2 (1.00)**	**1.00**	**1.00**
		1	B	BB	AA	AABB	AAB	2/4	2/2	1/3	**1/2 (0.50)**	**0.50**	**0.50**
		0		BB	AA	AABB	AA	2/4	2/2	0/2	**0/2 (0.00)**	**0.00**	**0.00**
2∶3	2∶3	4	BBBB	BB	AA	AAABB	AAABBBB	2/5	2/3	4/7	4/3	**2.00**	**2.00**
		3	BBB	BB	AA	AAABB	AAABBB	2/5	2/3	3/6	3/3	**1.50**	**1.50**
		2	BB	BB	AA	AAABB	AAABB	2/5	2/3	2/5	2/3	**1.00**	**1.00**
		1	B	BB	AA	AAABB	AAAB	2/5	2/3	1/4	1/3	**0.50**	**0.50**
		0		BB	AA	AAABB	AAA	2/5	2/3	0/3	0/3	**0.00**	**0.00**
3∶2	2∶3	4	BBBB	BB	AA	AABBB	AAABBBB	3/5	3/2	4/7	4/3	0.89	**2.00**
		3	BBB	BB	AA	AABBB	AAABBB	3/5	3/2	3/6	3/3	0.67	**1.50**
		2	BB	BB	AA	AABBB	AAABB	3/5	3/2	2/5	2/3	0.44	**1.00**
		1	B	BB	AA	AABBB	AAAB	3/5	3/2	1/4	1/3	0.22	**0.50**
		0		BB	AA	AABBB	AAA	3/5	3/2	0/3	0/3	0.00	**0.00**

The allele B is the B allele, and the values for various parameters are the expected values.

N-H mixture or N-H mix, the mixture of normal and H-mole DNAs; C-H mixture or C-H mix, the mixture of cancer and H-mole DNAs; N-H mix ratio, the ratio of normal and H-mole DNA amounts in the N-H mixture; C-H mix ratio, the ratio of cancer and H-mole DNA amounts in the C-H mixture; Copy Number, the copy number in cancer; Cancer Genotype, the genotype in cancer-sample DNA; Normal genotype, the genotype in normal-sample DNA; N-H mix genotype, the genotype in the N-H mixture; C-H mix genotype, the genotype in the C-H mixture; BAF_NH_, the ratio of B allele and total allele amounts (or B allele frequency) in the N-H mixture; AR_NH_, the ratio of B allele and A allele amounts (or allelic ratio) in the N-H mixture; BAF_CH_, the ratio of B allele and total allele amounts (or B allele frequency) in the C-H mixture; AR_CH_, the ratio of B allele and A allele amounts (or allelic ratio) in the C-H mixture; ARR, the ratio of AR_CH_ and AR_NH_, or the AR ratio; nARR, the ARR values that are divided by the median ARR value, or normalized ARR.

The median ARR value (underlined) was used for normalization.

**Figure 3:**
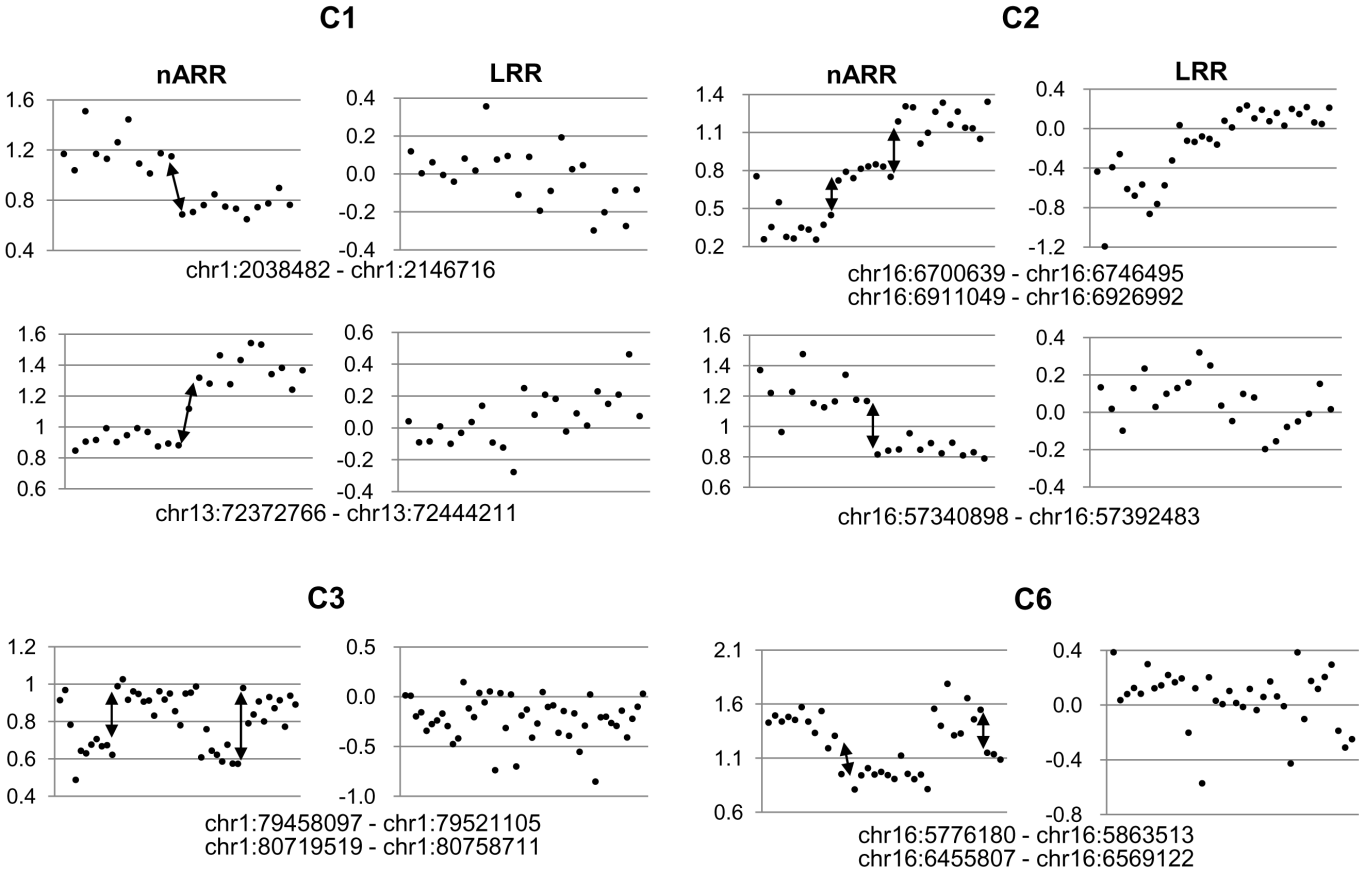
Copy-number changes at CNBs according to nARR values or LRRs. The copy-number statuses at nine CNBs in four primary colon cancer samples are shown. The CNBs were apparent with the nARR values, but the LRRs showed no clear breakpoints at most sites. The arrows indicate the CNBs employed for capture probes; at all of the CNBs marked by an arrow, the PCR-amplifiable tumor-specific rearrangements were successfully identified by TNGS-CNB.

The representative copy-alteration patterns analyzed based on the LRR, nAR_CH_, and nARR are shown in Figure 2B. The CNBs could be defined better with the nARRs than with the LRRs, as shown in Figure 3. At all of the CNBs indicated in the figure, specific rearrangements were later confirmed. Therefore, the nARR, as calculated from the cSNP microarray, was employed for further analysis. At least five CNBs from each sample were selected. In all of the nine samples, 91 breakpoints (Table S1) were selected, based on the nARR values for the seven primary colon cancers and the nAR_CH_ values for the two cancer cell lines.

### Analysis of Rearranged Sequences after Sequence-Capture Array

To enrich the rearranged sequences, targeted paired-end NGS was performed, and a total 5.7 Gb of sequence reads was obtained. The sequencing coverage was 180–475x for each sample, for an average coverage of 294.1x.

After alignment and filtering of the sequence reads using publicly available software programs [20], [21], 295 rearranged sequences within the 91 CNBs were analyzed (Table S1). After removal of the rearranged sequences outside of the sample-specific CNBs for each sample, 32 remained. After the removal of an additional nine rearranged sequences that were found in repetitive sequences, a final total of 23 rearranged sequences remained (Table 2).

**Table 2:** PCR-amplified rearrangements identified by TNGS-CNB.

Sample	ID	Copy-numberbreakpoint region		TNGS					Amplifi-ed in tumor	Amplifi-ed in normal
				Read end 1		Read end 2		Predicted cDNA		
		Chr.*	Position (in nucleotides)	Chr.*	Position	Chr.*	Position			
C1	C1–1	1	32620989–32670780	1	Intergenic DNA	1	C1orf86 intron	Exon 1–7 deletion of C1orf86	Y	
	C1–2	13	72372766–72444211	13	DACH1 intron	13	DACH1 intron	Partial exon 1 and exon 2–3 deletion of DACH1	Y	
C2	C2–1	16	6700639–6746495	16	RBFOX1 intron	16	RBFOX1 intron	Exon 3 deletion of RBFOX1	Y	
	C2–2	16	6911049–6926992	16	RBFOX1 intron	16	RBFOX1 intron	Exon 3 deletion of RBFOX1	Y	
	C2–3	16	57340898–57392483	16	Intergenic DNA	21	Intergenic DNA		Y	
C3	C3–1	1	79458097–79521105	1	Intergenic DNA	1	Intergenic DNA		Y	
	C3–2	1	80719519–80758711	1	Intergenic DNA	2	ANXA4 intron	Exon 2–13 deletion of ANXA4	Y	
C4	C4–1	5	3764835–3856153	5	Intergenic DNA	5	Intergenic DNA		Y	
	C4–2	7	54247350–54264207	7	Intergenic DNA	7	FKBP9L intron		Y	Y**
C5	C5–1	8	38120026–38193268	8	WHSC1L1 intron	17	Intergenic DNA	Exon 1–12 deletion of WHSC1L1	Y	
C6	C6–1	16	5776180–5863513	16	Intergenic DNA	16	RBFOX1 intron	Exon 1 deletion of RBFOX1	Y	
	C6–2	16***	6455807–6569122	16	RBFOX1 intron	16	RBFOX1 intron		N	
	C6–3			16	RBFOX1 intron	16	RBFOX1 intron	Exon 3 deletion of RBFOX1	Y	
C7****	C7–1	3	60448421–60459041	3	FHIT intron	3	FHIT intron	Exon 5 deletion of FHIT	Y	
	C7–2	5	27040172–27058600	5	Intergenic DNA	5	Intergenic DNA		Y	
	C7–3	9	121112114–121136985	9	Intergenic DNA	9	ASTN2 intron	Exon 4–22 deletion of ASTN2	Y	
	C7–4	16	83284155–83293878	16	CDH13 intron	16	CDH13 intron	Exon 3–5 deletion of CDH13	Y	
C8****	C8–1	16	6700639–6746495	16	RBFOX1 intron	16	RBFOX1 intron	No deletion of RBFOX1	Y	
	C8–2	8	128601683–128606353	8	Intergenic DNA	8	Intergenic DNA		Y	
	C8–3	8	129746645–129857169	8	Intergenic DNA	8	Intergenic DNA		Y	
	C8–4	12	27552182–27572094	12	ARNTL2 intron	12	Intergenic DNA	Exon 15–17 deletion of ARNTL2	Y	
	C8–5	16	82855099–82866517	16	CDH13 intron	7	Intergenic DNA		Y	Y**
	C8–6	22	31618708–31645509	22	LIMK2 intron	22	PRR14L intron		Y	

*Chromosome number.

**Two rearrangements were amplified also in normal samples, indicating that these are constitutive genomic rearrangements.

***Two candidate rearrangements were analyzed by next-generation sequencing in the same region as sample C6, but only one was amplified by PCR.

****Samples C7 and C8 are the COLO205 and SW620 cancer cell lines, respectively.

ID, identification number.

### Confirmation of Rearranged Sequences and Tumor Specificity

**Figure 4:**
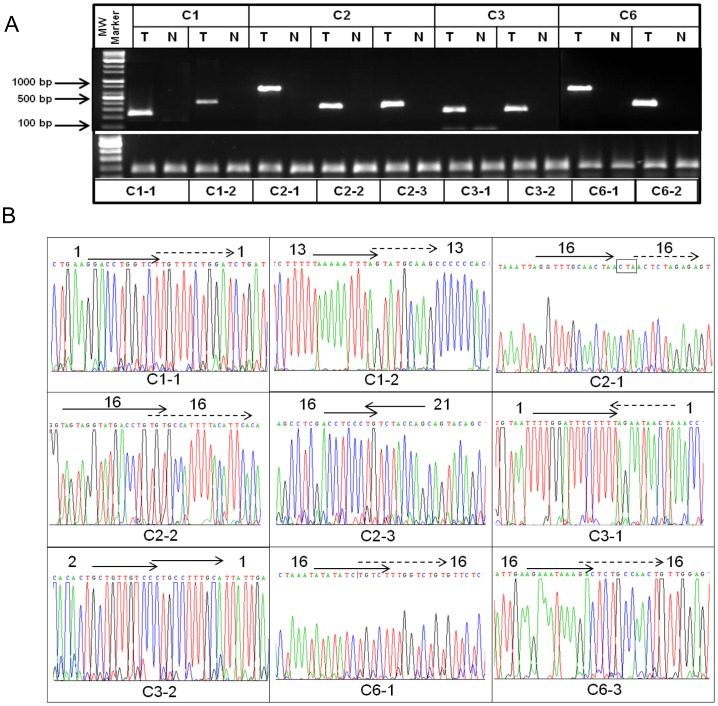
Tumor-specific rearrangements identified by TNGS-CNB in samples C1, C2, C3, and C6. A. Tumor-specific PCR amplifications at rearrangement sites. T, tumor; N, normal. In lower panel, *IGF1* amplification was used as a positive control. B. Sequencing data in rearranged sequences identified by TNGS-CNB. The arrow direction is from the telomeric side of the chromosomal short arm toward the telomeric side of the long arm. A dotted arrow is closer to the telomeric side of the chromosomal long arm than a lined arrow. The numbers beside the arrows are the chromosome numbers. The CNBs (C1–1, C1–2, C2–1, etc.) are the same as in Table 2.

To confirm the tumor-specificity of the rearrangements, PCR was performed on the rearranged sequences identified by targeted sequencing, in both the tumors and the corresponding normal tissues. With regard to the two cancer cell lines meanwhile, for which no controls are available, PCR was performed in 10 control whole-blood-DNA samples. The results showed that in both the tumors and corresponding normal tissues, two rearranged sequences were not amplified (Table 2), suggesting non-specific signals from targeted sequencing. Another two failed to show tumor-specific amplification, which indicated that they were constitutional genomic rearrangements (Table 2). Thus, total tumor-specific amplification was shown at 19 sites (Figs. 4A and S1A). Finally, all of the rearranged sequences were reconfirmed by Sanger sequencing (Figs. 4B and S1B).

Although the candidate CNBs had been selected randomly, not based on the locations of specific genes, intragenic deletions were found at 13 rearrangement sites, and there were exonal deletions in 12 of the 19 rearrangements (Table 2). The rearranged genes showing exonal deletions, namely *FHIT* [22], *CDH13* [23], *DACH1* [24], and *RBFOX1* [25], had been reported as deleted or inactivated in cancer. However, partial exonal deletions in those genes have not been widely studied. In the present results, there were frequent partial intragenic *RBFOX1* deletions in three cases (Table 2), though their biological significance was not clear. An intragenic *WHSC1L1* rearrangement containing a deletion in exon 1–12 also was detected, but once again, the biological significance was uncertain, because *WHSC1L1* is known as an oncogene. Intragenic rearrangements in C1orf86, *ANXA4*, *ASTN2*, and *ARNTL2* also were found (Table 2).

## Discussion

In the present study, we evaluated a method of TNGS-CNB for obtainment of information on rearranged ends in solid tumors, in combination with a novel cSNP microarray employing H-mole DNA as a competitor to refine the CNB regions. In our analysis, 19 tumor-specific PCR-amplifiable rearrangements from 91 CNBs were identified in seven primary colon cancers and two colon cancer cell lines, COLO205 and SW620: there was one rearrangement in 88.9% of cases (8/9), and two in 66.7% of cases (6/9). Notably, the rate of positive identification of PCR-amplifiable rearrangements was remarkably high (82.6%, 19/23), which relieved the burden of any validation procedure. Our overall data indicates the clinical-application potential of TNGS-CNB for obtainment of information on rearranged ends in solid tumors.

Although TNGS-CNB’s obtainment of only limited rearrangement information is a drawback, it offers several advantages over whole-genome NGS for solid-tumor monitoring. TNGS-CNB does not require extremely complicated and time-consuming bio-informatics procedures, owing to the small handling volume of targeted sequences. Moreover, it requires much less time for validation, because most of the candidate sites selected by publicly available software programs were positively identified as PCR-amplifiable tumor-specific rearrangements (82.6%, 19/23). TNGS-CNB can also be applicable to archival tissues, as formalin-fixed paraffin-embedded tissues have successfully been used for targeted NGS [26] and SNP microarrays [27]. Its cost, however, is similar to or less than that for whole-genome NGS, when calculated based on the currently available price in Korea. The cost of a cSNP array per case is about $900 (3 array analyses per sample). Since the cost of targeted NGS for 16 samples is about $10,000, the cost per case is about $700. So, the total cost of TNGS-CNB per case is about $1,600, which, again, is similar to or less than that of whole-genome NGS (about $2,500 for 30x read depth with Illumina HiSeq X Ten). Although two PCR-amplifiable tumor-specific rearrangements were identified in only 66.7% of the cases in the present study, this success rate will be improved by increasing the number of candidate capture sites in CNBs. When hotspot CNBs in solid tumors become available from the large amount of whole-genome NGS data currently being processed, especially with x100 read depth, TNGS-CNB will be effective for clinical application in solid tumors with a focused panel specific for various cancer subtypes. However, further validation of TNGS-CNBs on large clinical cancer cohorts is needed.

Even though the CNBs were randomly selected, intragenic rearrangements were identified in most of the confirmed rearrangements (68%, 13/19). Among these rearrangements, 12 contained small exonal deletions, and most of the affected genes, including *FHIT* [22], *CDH13* [23], *DACH1* [24], and *RBFOX1* [25], have been reported as tumor suppressors or as deleted in cancer, suggesting their active role in tumorigenesis. Among the small intragenic exonal deletions, five intragenic rearrangements of *RBFOX1* were found in three cases, suggesting that small intragenic rearrangements are recurrent in solid tumors. Frequent deletion of *RBFOX1* in colon cancer, reported in an earlier study [25], supports its active role in tumorigenesis. In addition to tumor suppressors, a rearrangement in an oncogene, *WHSC1L1*, was found in the present study. A similar *WHSC1L1* intragenic rearrangement was reported in a previous study [28], though deletion of the *WHSC1L1* oncogene can hardly explain the tumorigenic process right now, suggesting the need for further elucidation of the biological significance of *WHSC1L1* intragenic deletion. And although intragenic rearrangements have not been paid much attention, their high detection rate at randomly selected CNBs, in the present study, inspires the expectation that further investigation with this technology will reveal both additional intragenic rearrangements and their clinical and biological significance in cancer.

Whereas the capture technology was quite effective for identification of rearranged sequences in solid tumors, the capture efficiency of rearranged sequences was low, due to several factors. First, as already recognized [29], [30], routine exclusion of repetitive sequences in capture probe design can be a factor, because repetitive sequences have been posited as the major sites of genomic rearrangements [31], [32], [33], [34], and most of them cannot be amplified tumor-specifically with PCR technology. Second, there is the issue of cancer tissue contamination by normal cells, and indeed, the importance of cancer cell proportion to the detection of molecular changes is well understood [35]. Third, small genomic DNA fragments employed for targeted capture also can negatively impact capture efficiency. When the genomic DNA fragment for targeted capture is larger, the binding efficiency between the probe and the fragment will be higher in rearranged fragments, because there are more available bases for hybridization with probes without affecting the binding efficiency (especially when the target capture size, about 200 bp, is less than two times larger than the capture probe size, about 120 bp). It seems, in any case, that further study on capture efficiency optimization for rearranged sequences will be necessary.

It should also be noted that some important parameters in the proposed TNGS-CNB method, such as probe synthesis, CNB site determination, and target sequence data analysis, are carried out by expert companies; consequently the turn-around time (about 6 weeks for probe synthesis) is a little longer, but it will be easier to standardize the experimental process when large numbers of samples are processed. With the advancement of targeted sequencing methods in the near future, the total turn-around time will also drop significantly. Another consideration is that the data processing for TNGS-CNB is very simple and straightforward compared with whole-genome NGS sequencing.

Competitive PCR methodology utilizing SNP alleles has been reported to measure nucleotide copy numbers with superb sensitivity [36], [37]. We recently showed that a modified version of competitive PCR, mrcPCR, could detect various copy-number alterations and variations within a short assay time, with a small sample requirement, and with high reliability [38]. However, its principle had not, prior to the present study, been applied to microarray technology. Thus, we applied it specifically to SNP microarray technology employing complete H-mole genomic DNA (the SNPs of which are all homozygous) [39] as a competitor. In the results, we found that CNBs were much more easily narrowed down with our cSNP microarray technology than with the conventional SNP microarray utilizing LRRs. The commercially available HumanCytoSNP-12 that we utilized for our cSNP microarray, however, yielded only a limited number of informative alleles: about 30–40 K (10–14%) informative alter homozygote alleles out of 290 K SNPs for each sample. The current cSNP microarray technology could be more useful when employed using a customized SNP microarray designed with alleles rare in the general population among alleles in H-mole DNA. Significantly, when a cSNP microarray consists of alleles of <0.3 frequency in the general population, at least 49% of SNPs can be informative.

In conclusion, we showed that TNGS-CNB, entailing CNB-region refinement using competitive SNP microarray technology, can be a useful means of obtaining information on PCR-amplifiable rearranged sequences in solid tumors: two or more PCR-amplifiable tumor-specific rearrangements were obtained in two-thirds of colon cancer cases in a relatively simple and cost-effective way. Further clinical validation studies on TNGS-CNB as a cancer-relapse and therapy-response monitoring tool applicable to solid tumors would be warranted.

## Supporting Information

Figure S1 **Tumor-specific rearrangements identified by TNGS-CNB in samples C4, C5, C7 and C8.**
(DOC)

Table S1 Copy-number breakpoints deduced from competitive SNP microarray.
(DOC)

Table S2 Primers for PCR verification of rearranged sequences.
(DOC)
