# Supplementary material for: Targeted Next-Generation Sequencing at Copy-Number Breakpoints for Personalized Analysis of Rearranged Ends in Solid Tumors
Source: PLoS One. 2014 Jun 17;9(6):e100089. doi: 10.1371/journal.pone.0100089 (PMC4061055; doi:10.1371/journal.pone.0100089)
Supplement: Figure S1 — Tumor-specific rearrangements identified by TNGS-CNB in samples C4, C5, C7 and C8. (DOC) [file pone.0100089.s001.doc]

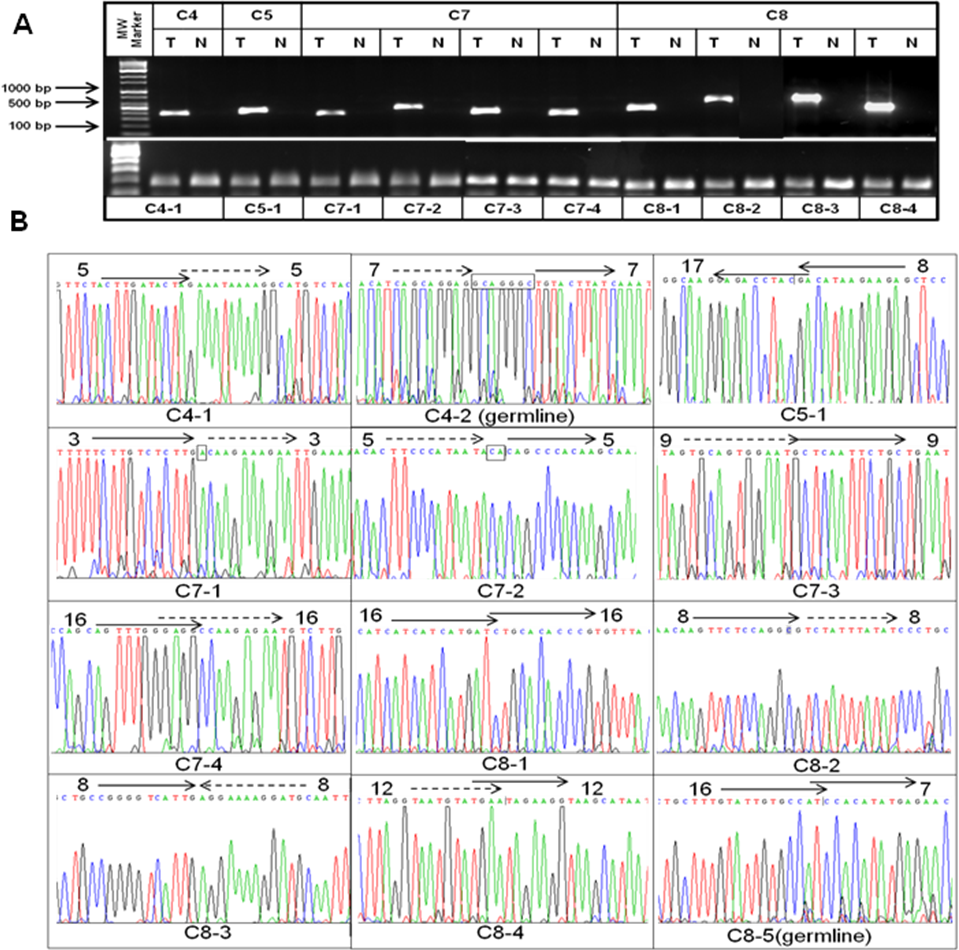


Figure S1. Tumor-specific rearrangements identified by TNGS-CNB in samples C4, C5, C7 and C8. A. Tumor-specific PCR amplifications at rearrangement sites. T, tumor; N, normal. In lower panel, *IGF1* amplification was used as a positive control. B. Sequencing data on rearranged sequences identified by TNGS-CNB. The arrow direction is from the telomeric side of the chromosomal short arm toward the telomeric side of the long arm. A dotted arrow is closer to the telomeric side of the chromosomal long arm than a lined arrow.
