# Supplementary material for: Targeted Next-Generation Sequencing at Copy-Number Breakpoints for Personalized Analysis of Rearranged Ends in Solid Tumors
Source: PLoS One. 2014 Jun 17;9(6):e100089. doi: 10.1371/journal.pone.0100089 (PMC4061055; doi:10.1371/journal.pone.0100089)
Supplement: Table S1 — Copy-number breakpoints deduced from competitive SNP microarray. (DOC) [file pone.0100089.s002.doc]

Table S1. Copy-number breakpoints deduced from competitive SNP microarray

| Sample | Chr. | Position (Nucleotide) | SNP | Size (kb) |
| --- | --- | --- | --- | --- |
| C1 | 1 | 2038482-2146716 | rs12410859-rs7512482 | 108.2 |
|  | 1 | 32620989-32670780 | rs16834897-rs2295116 | 49.8 |
|  | 3 | 175074859-175117882 | rs6771725-rs1862077 | 43.0 |
|  | 4 | 168576349-168582977 | rs6850404-rs17524463 | 6.6 |
|  | 6 | 162793774-162805900 | rs7752854-rs9365412 | 12.1 |
|  | 7 | 10952363-10974761 | rs12699164-rs218979 | 22.4 |
|  | 13 | 72372766-72444211 | rs9572793-rs4884967 | 71.4 |
|  | *16 | 7035798-7115040 | rs11077121-rs2881358 | 79.2 |
|  | 17 | 31141283-31147394 | rs2430988-rs34822561 | 6.1 |
|  | 20 | 15651637-15699639 | rs1362512-rs16996421 | 48.0 |
|  | 20 | 59173080-59271340 | rs846201-rs4812180 | 98.3 |
| C2 | 1 | 12467370-12483427 | rs11801678-rs12058577 | 16.1 |
|  | 3 | 57944993-57977182 | rs1675382-rs839248 | 32.2 |
|  | *16 | 6700639-6746495 | rs17665477-rs1544616 | 45.9 |
|  | 16 | 6911049-6926992 | rs11646704-rs2131226 | 15.9 |
|  | 16 | 19991934-20034746 | rs2883661-rs1350380 | 42.8 |
|  | 16 | 57340898-57392483 | rs1787781-rs4359426 | 51.6 |
|  | 20 | 15183643-15193682 | rs175223-rs17362002 | 10.0 |
|  | 21 | 30196654-30358161 | rs8131527-rs2245537 | 161.5 |
|  | 21 | 30030780-30115649 | rs2831951-rs11088088 | 84.9 |
| C3 | 1 | 79458097-79521105 | rs6685355-rs3795404 | 63.0 |
|  | 1 | 80719519-80758711 | rs12563768-rs17104602 | 39.2 |
|  | 16 | 7468479-7492774 | rs7190503-rs2109437 | 24.3 |
|  | 17 | 49856595-49915918 | rs1526189-rs202132 | 59.3 |
|  | 19 | 10103333-10116125 | rs2010448-rs2303099 | 12.8 |
|  | 19 | 57576664-57626098 | rs4435360-rs10423919 | 49.4 |
|  | 20 | 9734520-9738281 | rs2423462-rs16996383 | 3.8 |
|  | 21 | 33139776-33146571 | rs2833496-rs204750 | 6.8 |
|  | 21 | 35260231-35271623 | rs2073370-rs3761353 | 11.4 |
| C4 | 1 | 11111379-11169426 | rs12121344-rs2275525 | 58.0 |
|  | 3 | 3311470-3376571 | rs1153452-rs1499087 | 65.1 |
|  | 3 | 3792695-3850997 | rs1073657-rs12054065 | 58.3 |
|  | 5 | 24263840-24329414 | rs5007912-rs6452200 | 65.6 |
|  | 5 | 3764835-3856153 | rs13436137-rs9313059 | 91.3 |
|  | 6 | 162601471-162659816 | rs1954948-rs12203066 | 58.3 |
|  | 7 | 54247350-54264207 | rs7799840-rs11765237 | 16.9 |
|  | 7 | 55718151-55777865 | rs10259734-rs4563845 | 59.7 |
|  | 8 | 117778320-117838373 | rs10283122-rs10109496 | 60.0 |
|  | *16 | 6124881-6154979 | rs9927558-rs11077003 | 30.1 |
|  | 16 | 7115213-7168255 | rs2346254-rs1478691 | 53.0 |
| C5 | 1 | 20814272-20927904 | rs1152984-rs577042 | 113.6 |
|  | 1 | 1021165-1030315 | rs3737728-rs6687776 | 9.2 |
|  | 3 | 85618058-85663599 | rs1375555-rs6779752 | 45.5 |
|  | 3 | 85689893-85697364 | rs4507269-rs9810211 | 7.5 |
|  | 5 | 9716299-9762317 | rs1015855-rs16883522 | 46.0 |
|  | 8 | 38120026-38193268 | rs6981405-rs10101168 | 73.2 |
|  | 8 | 38803976-38940684 | rs6474513-rs4130393 | 136.7 |
|  | 10 | 7178745-7187565 | rs1886285-rs2762559 | 8.8 |
|  | 11 | 33779423-33849658 | rs1112841-rs6484673 | 70.2 |
|  | 11 | 34058476-34066871 | rs11032502-rs4497383 | 8.4 |
|  | 17 | 7213444-7285581 | rs4796399-rs2269762 | 72.1 |
|  | 17 | 66871950-66895229 | rs4148010-rs4968824 | 23.3 |
|  | 18 | 28597045-28656485 | rs276912-rs3910498 | 59.4 |
|  | 18 | 29286551-29319173 | rs1941930-rs12604302 | 32.6 |
| C6 | 1 | 106220779-106286066 | rs1340449-rs791004 | 65.3 |
|  | 16 | 5776180-5863513 | rs12597267-rs7198171 | 87.3 |
|  | *16 | 6124881-6255166 | rs9927558-rs10500334 | 130.3 |
|  | 16 | 6455807-6569122 | rs2795534-rs17540794 | 113.3 |
|  | *16 | 7035798-7143565 | rs11077121-rs17736941 | 107.7 |
| C7^†^ | 3 | 178275-195065 | rs4684051-rs10510184 | 16.8 |
|  | 3 | 60448421-60459041 | rs9809480-rs2734390 | 10.6 |
|  | 5 | 27040172-27058600 | rs7711992-rs6897759 | 18.4 |
|  | 5 | 27123043-27191458 | rs28716546-rs6880526 | 68.4 |
|  | 6 | 51110498-51142851 | rs4711968-rs9463680 | 32.4 |
|  | 6 | 138472153-138476781 | rs12190715-rs1883468 | 4.6 |
|  | 8 | 114908766-114948578 | rs7834762-rs6469494 | 39.8 |
|  | 9 | 120085926-120107794 | rs10983619-rs10818024 | 21.9 |
|  | 9 | 121112114-121136985 | rs7849366-rs17366047 | 24.9 |
|  | 12 | 125204122-125217028 | rs838853-rs12370375 | 12.9 |
|  | 16 | 83284155-83293878 | rs8053948-rs2113294 | 9.7 |
|  | 19 | 5684525-5717816 | rs35832694-rs2485279 | 33.3 |
| C8^†^ | 2 | 77468290-77479304 | rs6705164-rs3943682 | 11.0 |
|  | 3 | 60315458-60333199 | rs6762886-rs11920482 | 17.7 |
|  | 3 | 60918707-60937080 | rs9876711-rs9811973 | 18.4 |
|  | 6 | 100030787-100035599 | rs514769-rs685918 | 4.8 |
|  | 8 | 128601683-128606353 | rs13253038-rs13259282 | 4.7 |
|  | 8 | 128765830-128784147 | rs11992286-rs10112382 | 18.3 |
|  | 8 | 129746645-129857169 | rs6990777-rs13250924 | 110.5 |
|  | 12 | 27552182-27572094 | rs1562048-rs499675 | 19.9 |
|  | 15 | 85948459-85997996 | rs13379803-rs7178591 | 49.5 |
|  | *16 | 6700639-6746495 | rs17665477-rs1544616 | 45.9 |
|  | 16 | 7270332-7278634 | rs6500936-rs8059764 | 8.3 |
|  | 16 | 82855099-82866517 | rs2549147-rs10492861 | 11.4 |
|  | 22 | 31618708-31645509 | rs5749237-rs2106294 | 26.8 |
| C9 | 1 | 8839300-8868406 | rs7513880-rs12137865 | 29.1 |
|  | 1 | 48758458-48774515 | rs6684996-rs1608695 | 16.1 |
|  | 1 | 64140322-64173234 | rs1740402-rs1506399 | 32.9 |
|  | 1 | 80386090-80403167 | rs11162867-rs159665 | 17.1 |
|  | 7 | 155923366-155989419 | rs741828-rs10243418 | 66.1 |
|  | 8 | 130421318-130450096 | rs13259353-rs2217589 | 28.8 |
|  | 17 | 59974651-60007248 | rs11658522-rs12452094 | 32.6 |
|  | 20 | 22487988-22506050 | rs6082725-rs6048155 | 18.1 |

* Copy-number breakpoints at the same or similar site in two different samples.

^†^ Samples C7 and C8 are the COLO205 and SW620 cancer cell lines, respectively.
