# Supplementary material for: Targeted Next-Generation Sequencing at Copy-Number Breakpoints for Personalized Analysis of Rearranged Ends in Solid Tumors
Source: PLoS One. 2014 Jun 17;9(6):e100089. doi: 10.1371/journal.pone.0100089 (PMC4061055; doi:10.1371/journal.pone.0100089)
Supplement: Table S2 — Primers for PCR verification of rearranged sequences. (DOC) [file pone.0100089.s003.doc]

Table S2. Primers for PCR verification of rearranged sequences

| ID | Chr. | primer sequence 1 | primer sequence 2 |
| --- | --- | --- | --- |
| C1-1 | 1 | GGCATGACTGAAGGACCTGGTCTTGTTTCTG | GCTCCACTCAGGCAGCTCAAGTGA |
| C1-2 | 13 | GCATTTAATGCAAGATCACTCCATCATC | AGTGGAAAACACCCCTCAGAATAATGAGT |
| C2-1 | 16 | GAAGGGAAGTATCATTATCTCAGCAAGACAG | CCACTTTCATTTTCCATTTGTGTCCTGTAC |
| C2-2 | 16 | CATTCCAAACATACTGAGTCTTATTTGACAC | TAACAACTATCGGATGGGTTCACCTATTC |
| C2-3 | 16 | GCAAAGAAGCACTATGAACTTGGGATTGAG | CATGAAAGACTGAGGAATTGGATCAGATTAC |
| C3-1 | 1 | AGCAGATGAAAGCCAACTCGTAGTCA | CACTTGTAAACTGTTCTGGTAGCCATGAC |
| C3-2 | 1 | GATATGAAATGGGGTTGAATGAACAAGAACTG | GACTTTTGGGATTCCACAGGAAAGAATG |
| C4-1 | 5 | TCTGGGAGTATGATTTAACAAGCTCATTC | CAGTGATACATGATTGGTTTAATGCTGATTAG |
| C4-2 | 7 | CAGGATCAGCGGACAGGAATATGAAC | GACTAAAGGCTAATCAAGTGCTAGTAACACAG |
| C5-1 | 8 | GCCCTTACTTAGTTCCAGTCCTGACA | CTTGAGCTTCTTGGTGGCTACCATTATC |
| C6-1 | 16 | GGTGCCTCTACCTATTCAACAAATGTTAGTTC | CTCCAGGCAACGAAGGGTAGCAAGA |
| C6-2 | 16 | GTCCAGGTATATGCTGTGGTATCGTATTG | TGGTCTAGCTTGGGTCATTGGTAATCAC |
| C6-3 | 16 | AGCTACATGAGGGCTCTTGTTTTGTTAATC | CGCCAACTCCAAATGCCAGATAATC |
| C7-1 | 3 | TTGTAGGTGGCAGAAGGCAACAGAT | GCCACAGATATGAGGGACAAGTGACT |
| C7-2 | 5 | TAATGTTCTAGCCAGTTACTTTAACCTTCTG | TCTACCTGAGCTGACCTTCACAGTG |
| C7-3 | 9 | GATGCAAACTGAGCTTCTGCTGAAATAGAG | GAATTCCCTCTGCTTCAATGTTTTGA |
| C7-4 | 16 | GAGGCTGGAGTTATAGAGGCAGATGTTG | GGTGCTTGGCTTGCTACCACACTT |
| C8-1 | 16 | CCTCCTGGTTCCTCTCTGAATTACTTC | CCAGTTCCATCCATGTTGCTGTAAC |
| C8-2 | 8 | TCTGCCCTGGAGGAGTCCTTATCTG | CTCTTTCCATGTTGACCTCATTTTACTATTC |
| C8-3 | 8 | CACCTTACGGTTGTGAGTGAATGTTTGTC | CACTGGTTCTGCTGGAACTCTCTTTCAT |
| C8-4 | 12 | CCACTGAGTGTTCTTGGCACCCTTAGT | GAATAATCCCTGCACAGGTTGTTTAATACTG |
| C8-5 | 16 | TGCTTTGTATTGTGCCATCCACATATGAGAAC | CCATGTTCATTGCAGCACTATTCACA |
| C8-6 | 22 | GATGTCCCTGTCAACACAGCATTTGTTAC | AGGCAGAGATTGCAGTGAGCTGAGA |
| *IGF1* |  | TTCTCTAAATCCCTCTTCTGTTTGCTAAATC | GAGATGGGAGATGTTGAGAGCAATGT |
